# Oleuropein Attenuates Oxidative Stress in Human Trophoblast Cells

**DOI:** 10.3390/antiox12010197

**Published:** 2023-01-14

**Authors:** Andrea Pirković, Aleksandra Vilotić, Sunčica Borozan, Mirjana Nacka-Aleksić, Žanka Bojić-Trbojević, Milica Jovanović Krivokuća, Maurizio Battino, Francesca Giampieri, Dragana Dekanski

**Affiliations:** 1 Department for Biology of Reproduction, Institute for Application of Nuclear Energy (INEP), University of Belgrade, Banatska 31b, 11080 Belgrade, Serbia; 2 Department of Chemistry, Faculty of Veterinary Medicine, University of Belgrade, Bulevar Oslobođenja 18, 11000 Belgrade, Serbia; 3 International Joint Research Laboratory of Intelligent Agriculture and Agri-Products Processing, Jiangsu University, Zhenjiang 212013, China; 4 Dipartimento di Scienze Cliniche Specialistiche, Facoltà di Medicina, Università Politecnica delle Marche, 60131 Ancona, Italy; 5 Research Group on Food, Nutritional Biochemistry and Health, Universidad Europea del Atlántico, 39011 Santander, Spain

**Keywords:** oleuropein, trophoblast, oxidative stress, pregnancy

## Abstract

Olive-derived bioactive compound oleuropein was evaluated against damage induced by hydrogen peroxide in human trophoblast cells *in vitro*, by examining the changes in several markers implicated in oxidative stress interactions in the placenta. Trophoblast HTR-8/SVneo cells were preincubated with OLE at 10 and 100 µM and exposed to H_2_O_2_, as a model of oxidative stress. Protein and lipid peroxidation, as well as antioxidant enzymes’ activity, were determined spectrophotometrically, and DNA damage was evaluated by comet assay. iNOS protein expression was assessed by Western blot, while the mRNA expression of pro- and anti-apoptotic genes *BAX* and *BCL2* and transcription factor *NFE2L2*, as well as cytokines *IL-6* and *TNF α* were determined by qPCR. Oleuropein demonstrated cytoprotective effects against H_2_O_2_ in trophoblast cells by significantly improving the antioxidant status and preventing protein and lipid damage, as well as reducing the iNOS levels. OLE reduced the mRNA expression of *IL-6* and *TNF α,* however, it did not influence the expression of *NFE2L2* or the *BAX/BCL2* ratio after H_2_O_2_ exposure. Oleuropein per se did not lead to any adverse effects in HTR-8/SVneo cells under the described conditions, confirming its safety *in vitro*. In conclusion, it significantly attenuated oxidative damage and restored antioxidant functioning, confirming its protective role in trophoblast.

## 1. Introduction

Among the plants used in traditional and modern medicine, *Olea europaea* L. tree, popularly known as the olive tree, is one of the most widely used. Olive tree products are key components of the Mediterranean diet (MD) and are abundant in secondary compounds such as polyphenols, particularly flavonoids, with known benefits to human health [1]. One of the most important bioactive and functional food ingredients found in olive oil, olive fruits and olive leaves is phenolic secoiridoid oleuropein (OLE). It is best known for its antioxidant and hypotensive effects, but it is also associated with antiproliferative effects against cancer, anti-angiogenic, neuroprotective, anti-inflammatory and senolytic activity [2,3,4]. Recent studies provided insight into the disease-protective mechanism of OLE and explained many of its effects through oxidative stress modulation and antioxidant activity [5]. OLE showed an ability to suppress the production of reactive oxygen and nitrogen species (ROS and NOS, respectively) in *in vitro* biochemical assays, as well as in human cells, where it prevented excessive ROS generation [6]. It was also shown to attenuate oxidative stress (OS) by the modulation of the ERK/Nrf2 pathway-mediated signaling, with downstream consequences for a variety of cellular processes [7,8]. Although the many reported benefits of OLE provide ample rationale for its continued use, considerations about its possible interactions and side effects in certain vulnerable states, such as pregnancy, should be further examined. Pregnancy is a state of well-balanced OS, where certain amounts of ROS serve as signaling mediators in key placentation processes [9]. During the normal course of pregnancy, controlled redox signaling is pivotal, and a delicate balance between oxidant production and antioxidant protection is necessary at different stages of pregnancy. An imbalanced oxidative environment created by the overproduction of ROS disrupts these fine-tuned processes and could result in reproductive failure, such as fetal loss, intrauterine growth restriction (IUGR) or pregnancy disorders such as gestational diabetes mellitus (GDM) and preeclampsia (PE) [9,10]. Thus, antioxidant supplements have been proposed as a possible approach in the prevention and treatment of such disorders. The results of recent clinical trials with the MD and olive oil consumption during pregnancy, such as the St Carlos Study, ESTEEM trial, etc., showed that adherence to these diets provides health benefits in pregnant women and newborns and contributes to GDM prevention [11,12,13,14,15]. Furthermore, in the latest study by Minhas et al., the data from the Boston Birth Cohort showed that adherence to a MD was associated with a lower risk for PE development [16]. However, there are no human studies exploring the potential of OLE in pregnancy. The first step to examine its safety and possible benefits in pregnancy is to perform molecular and mechanistic studies that reflect OLE’s impact on the cellular networks involved in the regulation of the key pregnancy events. The initial steps and prerequisites for normal pregnancy establishment and maintenance are the successful implantation of the embryo into the maternal endometrium, and the subsequent invasion of extravillous trophoblast cells into the uterine stroma and maternal spiral arteries [17]. In the earliest stages of pregnancy, ROS-derived signals can act to stimulate or restrict the extent of trophoblast invasion and play an important role in proper placentation via modulation of trophoblast-cell-mediated adhesion, invasion and spiral artery remodeling. Increased OS can cause deregulation in these tightly regulated processes, which can culminate in placental dysfunction [18]. 

The aim of this research is to explore the impact of OLE on levels of OS in human trophoblast cells, by examining the changes in several OS-related markers (such as reduced glutathione, protein carbonyl levels, malondialdehyde, etc.) and molecular pathways that were found to be implicated in significant interactions between ROS and biological components in healthy and pathological pregnancy [19].

## 2. Materials and Methods

### 2.1. Cell Line

For the experiments performed in this research, the HTR-8/SVneo immortalized human extravillous trophoblast cell line (kindly provided by Dr Charles H. Graham, Queen’s, Kingston, ON, Canada) was cultured in a humidified incubator with 5% CO_2_ at 37 °C in complete culture medium (RPMI 1640 (Gibco, Life Technologies Ltd., Paisley, UK), supplemented with 10% fetal calf serum (Pan Biotech, Aidenbach, Germany) and 1% antibiotic/antimycotic solution (Capricorn Scientific GmbH, Ebsdorfergrund, Germany)).

### 2.2. Oleuropein Treatment Preparations 

A stock solution of oleuropein (Cas No. 32619-42-4, Extrasynthese, 100 mM in DMSO) was diluted in complete RPMI medium to reach final concentrations. In a preliminary experiment, increasing concentrations (1, 10, 25, 50, 100, 200 µM) of OLE in complete medium were tested for 24 h for cytotoxic effects on HTR-8/SVneo cells, using the 2,5-diphenyl-2H-tetrazolium bromide (MTT) assay on cell viability, according to the protocol described in Kostic et al. [20]. 

### 2.3. Evaluation of Oleuropein Effects against Cytotoxicity Induced by Hydrogen Peroxide in HTR-8/SVneo Cells

For the evaluation of the OLE against cytotoxic effects induced by hydrogen peroxide, HTR-8/SVneo cells were seeded at a density of 2 × 10^4^ cells/well in 96-well plates and allowed to attach overnight. On the following day, treatments with 10 and 100 μM OLE were added to the cells and incubated for 24 h, while control cells were treated with complete medium alone. After the 24 h treatment, cells were rinsed with phosphate-buffered saline (PBS) and 200 μM H_2_O_2_ in serum-free (SF) RPMI medium was added for 2 h. The concentration of 200 μM H_2_O_2_ was chosen as this concentration of H_2_O_2_significantly reduced cell viability compared to untreated cells. Next, the MTT assay was used to evaluate cell viability in HTR-8/SVneo cells, as described before in Kostic et al. [20]. The absorbance was measured at 570 nm using a microplate reader (ELx800, BioTek, Winooski, VT, USA). Average value for the control cells was set to 100%, and the results of treatments were presented as a percentage of control. Three independent experiments were performed in triplicate.

### 2.4. Investigation of Genotoxic and Antigenotoxic Effects of Oleuropein 

The genoprotective effect of OLE was evaluated using the alkaline comet assay, according to the treatment protocol described in Bruić et al. [21] and following the MIRCA guidelines on the comet assay [22]. The HTR-8/SVneo cells were seeded in 96-well plates (2 × 10^4^ cells/well) and, the following day, the treatments with 10 and 100 μM OLE in complete culture medium were added to the cells. After 24 h incubation with OLE, cells were rinsed, and genotoxic damage was induced by adding 30 µM H_2_O_2_ for 30 min in SF medium. This concentration of H_2_O_2_was selected because it was the lowest concentration that produced a significant increase in DNA damage compared to non-treated controls. After the induction of DNA damage, the cells were collected using 0.25% trypsin–EDTA solution and centrifuged at 300× *g* for 5 min, and the obtained pellet was used for the comet assay. In brief, the single-cell suspension was mixed with 0.7% low-melting-point agarose (Sigma Aldrich, St. Louis, MO, USA) and spread on previously pre-coated slides (with 1% normal-melting-point agarose, Sigma Aldrich, St. Louis, MO, USA). After the gel solidified, slides were placed in lysing solution, and a lysis step, followed by DNA unwinding and electrophoresis (at 25 V and 300 mA for 30 min), were performed as described in Bruić et al. [21]. Next, slides were neutralized with PBS and the staining of slides was performed with ethidium bromide (20 µg/mL). Comets were scored at a magnification of 40× on the Olympus BX 50 microscope (Olympus Optical Co., GmbH, Hamburg, Germany), equipped with a mercury lamp, HBO (50 W, 516–560 nm, Zeiss). Visual scoring and classification were performed as described previously by Collins and collaborators [23]. Each of the observed comets was given a value of 0, 1, 2, 3 or 4 (from undamaged, 0, to totally damaged, 4), and DNA damage was expressed in arbitrary units, calculated according to the equation (percentage of cells in class 0 × 0) + (percentage of cells in class 1 × 1) + (percentage of cells in class 2 × 2) + (percentage of cells in class 3 × 3) + (percentage of cells in class 4 × 4). Two replicate slides were analyzed for each treatment, and the scoring was performed on 100 randomly selected comets per slide. The entire experiment was repeated in triplicate.

### 2.5. Oxidative Stress Parameter Determination

To evaluate the OLE effects on OS parameters, HTR-8/SVneo cells were seeded in 6-well plates (5 × 10^5^ cells/well), left to attach overnight in complete medium and treated for 24 h with 10 and 100 µM OLE. The following day, treatments were removed, and the cells were exposed to 200 µM H_2_O_2_ for 2 h. Afterwards, the medium was collected and frozen at −20 °C until further analysis. The cells were rinsed 3 times with chilled PBS and cell lysates were prepared in ice-cold RIPA Buffer (Sigma Aldrich, St. Louis, MO, USA). Protein concentration in cell lysates was determined by using the bicinchoninic acid assay kit (BCA kit, Thermo Scientific, Rockford, IL, USA).

### 2.6. Antioxidant Enzyme Activity

Catalase (CAT) activity was determined by the UV–kinetic method in the presence of H_2_O_2_ [24]. Gluthatione peroxidase (GPx) activity was determined spectrophotometrically, according to the method of Günzler et al. [25]. Superoxide dismutase (SOD) activity was measured using the method based on the inhibition of epinephrine autoxidation [26]. The enzymes’ activity was expressed as enzyme activity per milligram of protein (U/mg protein).

### 2.7. Markers of Lipid Peroxidation and Oxidative Protein Damage

The method by Stocks and Dormandy [27] based on the reaction with thiobarbituric acid (TBA) was used to evaluate the concentration (nmol/mg protein) of MDA as a marker of lipid peroxidation. 

The extracellular lactate dehydrogenase (LDH) was determined through the reaction of the conversion of pyruvate to lactate and measured by the decrease in absorbance at 340 nm [28]. The activity of LDH is expressed as U/mL. 

Next, the reduced glutathione (GSH) was determined with the use of Ellman’s reagent (5,5′-dithio-bis(2-nitrobenzoic acid) [29]. Results were expressed as μmol of GSH per milligram of protein (μmol/mg protein). The reaction of carbonyl groups (CG) with 2,4-dinitrophenylhydrazine was measured spectrophotometrically at 365 nm, following the method of [30], and the results were expressed as nmol CG per milligram of protein (nmol/mg protein). 

All spectrophotometric measurements were performed on a Cecil CE 2021 UV/VIS spectrophotometer (Cecil Instruments Ltd., Milton, UK).

Finally, a colorimetric method with Griess reagent was employed for the quantitative analysis of nitrite (NO_2_^−^) concentrations, using the Guevara et al. method [31]. Absorbance was read on an ELISA reader at 540 nm (Plate reader, Mod. A1, Nubenco Enterprises, ICN, Paramus, NJ, USA). The results were expressed in μmol of NO_2_^−^ per milligram of protein (μmol/mg protein). 

### 2.8. Western Blot Analysis

Treated cells were lysed in RIPA buffer and incubated on ice for 30 min on a shaker to complete cell lysis, followed by centrifugation at 12,000× *g* for 20 min. Aliquots of lysates were kept at −80 °C until analysis. SDS–polyacrylamide gel electrophoresis was performed, and target protein levels were determined by Western blot analysis using antibodies for iNOS (ab3523, source: rabbit, 1:500, Abcam, Cambridge, UK) and visualized using enhanced chemiluminescence (ECL) (SERVA Electrophoresis GmbH, Heidelberg Germany) according to a method described elsewhere [32]. TL120 software was used to analyze the optical density of the protein bands and the values were normalized to the optical density of GAPDH.

### 2.9. Quantitative PCR Analysis

Total RNA was isolated from treated HTR-8/SVneo cells using TRI reagent solution (Thermo Fisher Scientific Baltics, Vilnius, Lithuania). First-strand cDNA was synthesized from 1 μg of total RNA, using 0.5 μg of Oligo(dT) 12–18 primers (Thermo Fisher Scientific Baltics, Vilnius, Lithuania), 250 μM of each dNTP and 200U of RevertAid reverse transcriptase (Thermo Fisher Scientific Baltics, Vilnius, Lithuania). Real-time PCR was performed as described in [33] using the 7500 Real-Time PCR System (Applied Biosystems, Carlsbad, CA, USA). Expression levels of *BCL2*, *BAX, NFE2L2 (Nrf2), IL-6 and TNFα* were normalized to the housekeeping gene *GAPDH*. Calculations were made by the comparative ΔΔCt method [34].

The sequences of specific primers were as follows: *BCL2* F: AGTTCGGTGGGGTCATGTGT; *BCL2* R: GGAGAAATCAAACAGAGGC; *BAX* F: TTGCTTCAGGGTTTCATC; *BAX* R: CACTCGCTCAGCTTCTTG; *NFE2L2* F: AGTGGATCTGCCAACTACTC; *NFE2L2* R: CATCTACAAACGGGAATGTCTG; *GAPDH* F: GAAGGTGAAGGTCGGAGT; *GAPDH* R: GAAGATGGTGATGGGATTTC; *IL6* F: GGTACATCCTCGACGGCATCT; *IL6* R: GTGCCTCTTTGCTGCTTTCAC; *TNF* F: CCCAGGCAGTCAGATCATCTT; *TNF* R: TCTCAGCTCCACGCCATT.

### 2.10. Statistical Analysis

One-way analysis of variance (ANOVA) with Tukey post-hoc analysis was used to evaluate the differences (α = 0.05) in treatments vs. control, after the data passed the normality test. All results were expressed as mean + standard error of the mean (mean + SEM). GraphPad Prism 6.0 (GraphPad Software, Inc., San Diego, CA, USA) was used for statistical analysis and *p* < 0.05 was considered significant. 

## 3. Results

### 3.1. Cytotoxicity of Oleuropein in HTR-8/SVneo Cells

**Figure 1:**
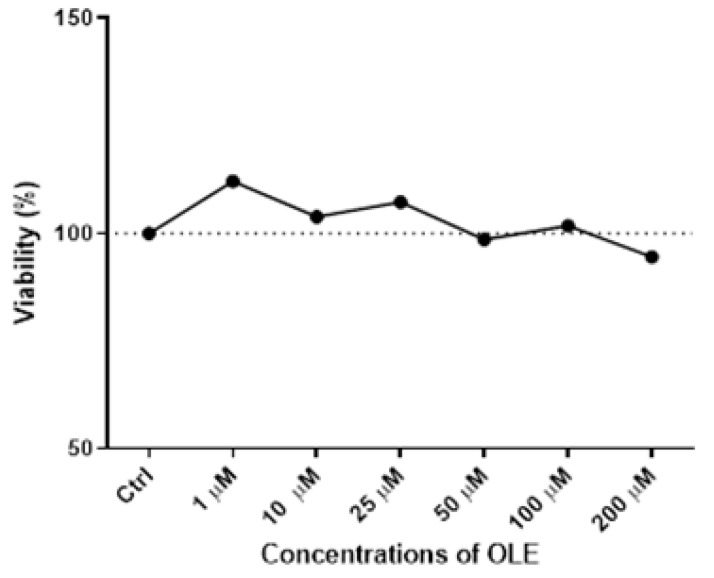
Cytotoxicity of oleuropein (OLE) in HTR-8/SVneo cells after 24 h treatment with a range of concentrations (1, 10, 25, 50, 100 and 200 µM).

The results of the MTT assay following the 24 h incubation with increasing OLE concentrations ranging from 1 µM to 200 µM in HTR-8/SVneo cells demonstrated a lack of cytotoxicity for the concentrations below 200 µM (Figure 1).

It could be observed that with the increase in OLE concentration, there was discrete cytotoxicity at the highest concentration of 200 µM, but without a statistically significant difference compared to control cells. Based on the obtained results (Figure 1), the concentration of 100 µM of OLE was chosen for testing high-dose OLE effects, as it was the highest concentration of OLE that did not influence cell viability. Accordingly, 10 µM of OLE was chosen for investigating low-concentration OLE effects on HTR-8/SVneo cells in the present study.

### 3.2. Oleuropein’s Cytoprotective Effect in H_2_O_2_-Treated HTR-8/SVneo Cells

**Figure 2:**
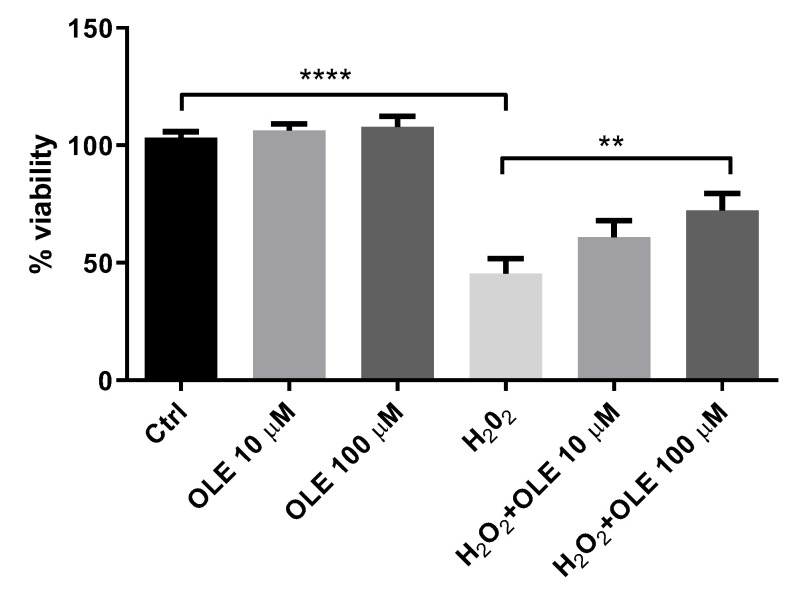
Cytoprotective effect of oleuropein (OLE) on H_2_O_2_-induced damage in HTR-8/SVneo cells. The data are expressed as mean + SEM. ** *p* < 0.01, **** *p* < 0.0001.

The cell viability of HTR-8/SVneo cells preincubated with 10 and 100 µM OLE was estimated by MTT assay after 2 h exposure to 200 µM H_2_O_2_ (Figure 2). It could be observed that 24 h pre-treatment of cells with 10 µM and 100 µM OLE did not influence cell viability compared to control cells. Among HTR-8/SVneo cells exposed to H_2_O_2_ alone, the viability was decreased to 45% versus non-treated controls. The pre-treatment of cells with 10 and 100 µM OLE prior to exposure to H_2_O_2_ showed a significant cytoprotective effect, i.e., it increased the percentage of viable cells (60.9% and 72.2%, respectively) compared to the cells incubated with H_2_O_2_ alone (45%).

### 3.3. Genotoxic and Antigenotoxic Properties of Oleuropein in H_2_O_2_-Treated HTR-8/SVneo Cells

**Figure 3:**
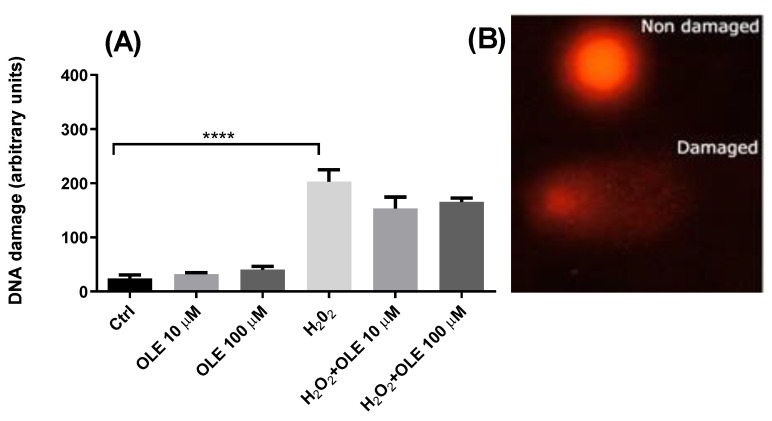
(**A**) Genotoxic potential of oleuropein (OLE) and antigenotoxic effects against H_2_O_2_-induced damage in HTR-8/SVneo cells. The data are expressed as mean + SEM. **** *p* < 0.0001. (**B**) Appearance of comets without DNA damage (Non damaged) and with DNA damage (Damaged).

The genotoxic potential of OLE was evaluated by incubating the HTR-8/SVneo cells for 24 h with 10 µM and 100 µM OLE. The results showed that, at the two examined concentrations, OLE did not induce any DNA damage compared to control cells exposed to medium alone (Figure 3). In order to evaluate the genoprotective potential of OLE, a model of OS-induced DNA damage was established by exposing the HTR-8/SVneo cells to 30 µM H_2_O_2_ for 30 min in SF RPMI medium. The treatment with H_2_O_2_ alone produced a significant increase in DNA damage compared to non-exposed controls. Pre-incubation of cells with OLE at concentrations of 10 µM and 100 μM, prior to H_2_O_2_ treatment, reduced the levels of H_2_O_2_-induced DNA damage; however, it did not exert statistically significant antigenotoxic effects.

### 3.4. The Effects of Oleuropein on Oxidative Stress Parameters in H_2_O_2_-Treated HTR-8/SVneo Cells

**Figure 4:**
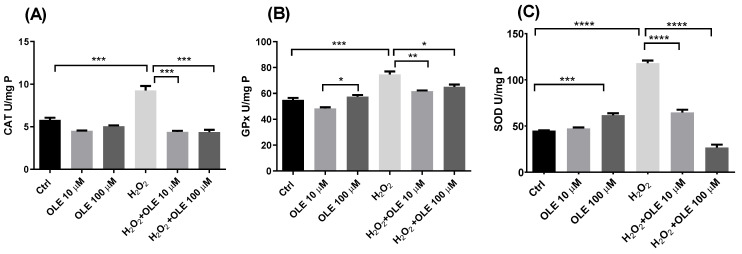
The effect of oleuropein (OLE) on activity of antioxidant enzymes in H_2_O_2_-exposed HTR-8/SVneo cells. The investigated parameters include catalase (CAT) activity (**A**), gluthatione peroxidase (GPx) activity (**B**) and superoxide dismutase (SOD) activity (**C**). The data are expressed as mean + SEM. * *p* < 0.05, ** *p* < 0.01, *** *p* < 0.001, **** *p* < 0.0001.

The alterations in the levels of antioxidant enzymes’ activity following the 24 h pre-incubation with OLE and subsequent exposure to H_2_O_2_ in HTR-8/SVneo cells are represented in Figure 4. It could be observed that OLE per se at concentrations of 10 µM and 100 µM did not induce changes in the total activity of enzyme CAT compared to non- treated controls (Figure 4A). The treatment of cells with 200 µM H_2_O_2_ only produced a significant increase in CAT activity. Further, in cells pre-treated with 10 µM and 100 µM OLE and incubated with H_2_O_2_, the activity of CAT was significantly decreased in both treatments in relation to the cells exposed to H_2_O_2_ alone. Furthermore, the observed differences were statistically significant for both OLE concentrations. Similar results could be seen for GPx and SOD activity, where OLE at 10 µM alone did not alter enzymes’ activity, while OLE at 100 µM produced an increase in both GPx and SOD activities (Figure 4B,C). Hydrogen peroxide treatment produced a significant rise in GPx and SOD activity compared to non-treated controls. Pre-incubation with 10 µM and 100 µM OLE provided a significant reduction in GPx activity following H_2_O_2_ exposure, compared to the treatment with H_2_O_2_ alone (Figure 4B). Both concentrations of OLE showed similar efficiency in decreasing the H_2_O_2_-induced GPx activity. The results of SOD activity in cells pre-incubated with OLE at 10 and 100 µM and exposed to H_2_O_2_ showed a concentration-dependent reduction in SOD activity compared to the high levels observed in the H_2_O_2_ treatment alone (Figure 4C). 

**Figure 5:**
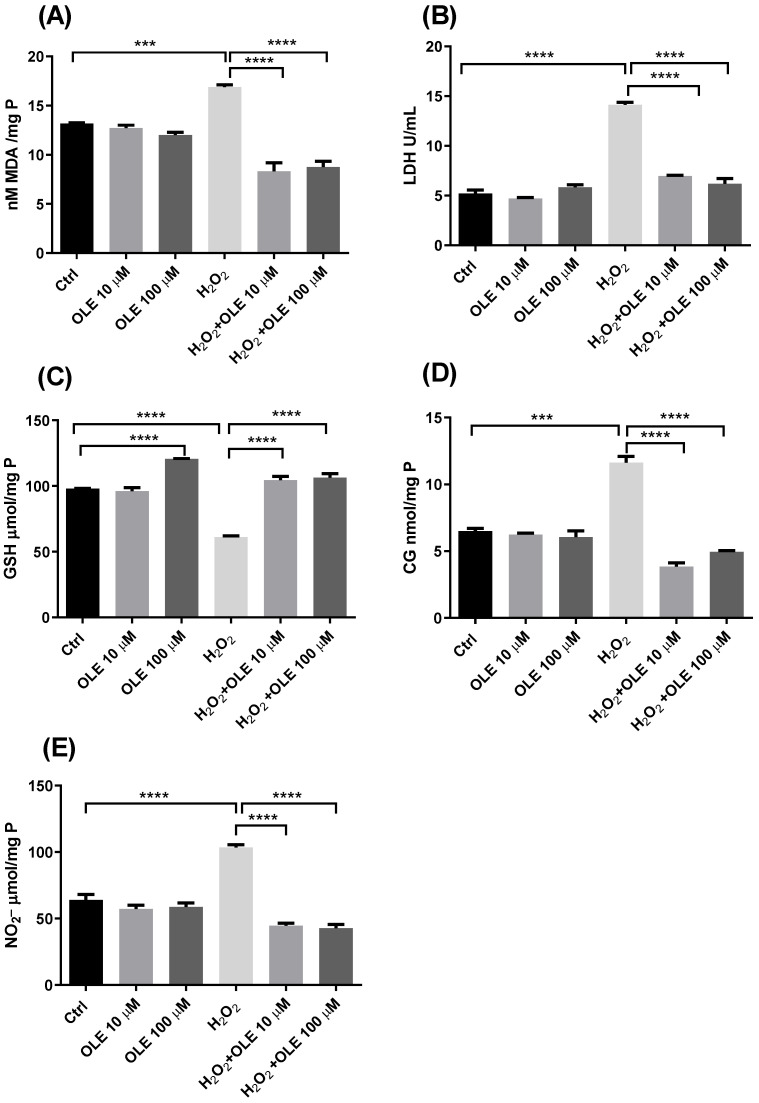
The effect of oleuropein (OLE) on lipid peroxidation and protein damage in H_2_O_2_-exposed HTR-8/SVneo cells. The investigated parameters include malondialdehyde (MDA) concentration (**A**), extracellular lactate dehydrogenase (LDH) activity (**B**), reduced glutathione (GSH) concentration (**C**), protein carbonyl group (CG) concentration (**D**) and nitrite (NO_2_^−^) concentration (**E**). The data are expressed as mean + SEM., *** *p* < 0.001, **** *p* < 0.0001.

The results of the concentration of MDA as a marker of lipid peroxidation are indicated in Figure 5A. While incubation with the two examined concentrations of OLE did not alter the level of MDA, exposure of cells to H_2_O_2_ significantly increased the rate of lipid peroxidation compared to non-treated controls. The cells pre-incubated with OLE and treated with H_2_O_2_ showed reduced MDA concentrations compared to the cells exposed to H_2_O_2_ alone. Both concentrations of 10 µM and 100 µM OLE provided statistically significant reductions in lipid peroxidation (Figure 5A). The results of extracellular LDH activity presented in Figure 5B showed that the treatment of cells with 10 µM and 100 µM OLE did not lead to membrane damage or the release of LDH extracellularly. In the cells exposed to H_2_O_2_ only, there was a significant increase in LDH activity compared to non-exposed controls. The pre-treatment with OLE exhibited protective effects and prevented LDH release after exposure to H_2_O_2_. This effect was similar and statistically significant for both OLE concentrations used in this study (Figure 5B).

The GSH content analysis shown in Figure 5C indicated that the treatment of cells with OLE 100 µM alone can provide a significant rise in GSH concentration, increasing the antioxidant capacity of HTR-8/SVneo cells. On the other hand, when the cells were exposed to H_2_O_2_, a significant decrease in GSH was observed compared to the non-treated control cells. Further, after the pre-treatment with OLE and the subsequent H_2_O_2_ challenge, an increase in GSH concentration was obtained, whereas both treatments with 10 µM and 100 µM OLE showed the significant recovery of the GSH concentration compared to H_2_O_2_ treatment alone (Figure 5C). Measurement of CG concentrations and NO^−^_2_, as markers of protein damage, in the cells treated with OLE and/or H_2_O_2_ are presented in Figure 5D,E. The results indicated that the 24 h treatment with OLE did not alter the levels of protein carbonylation or concentration of NO_2_^−^, whereas H_2_O_2_ significantly increased both CG and NO_2_^−^. Moreover, following the pre-incubation of cells with OLE and subsequent exposure to H_2_O_2_, the CG and NO_2_^−^ concentrations were significantly reduced and reached the values seen in the non-exposed controls. Both 10 µM and 100µM concentrations of OLE, used in the pre-treatment, showed similar efficiency in preventing CG and NO_2_^−^ formation after H_2_O_2_ exposure, with statistically significant difference compared to the treatment with H_2_O_2_ alone.

### 3.5. The Effect of Oleuropein on Inducible Nitric Oxide Synthase (iNOS) in H_2_O_2_-Exposed HTR-8/SVneo Cells

**Figure 6:**
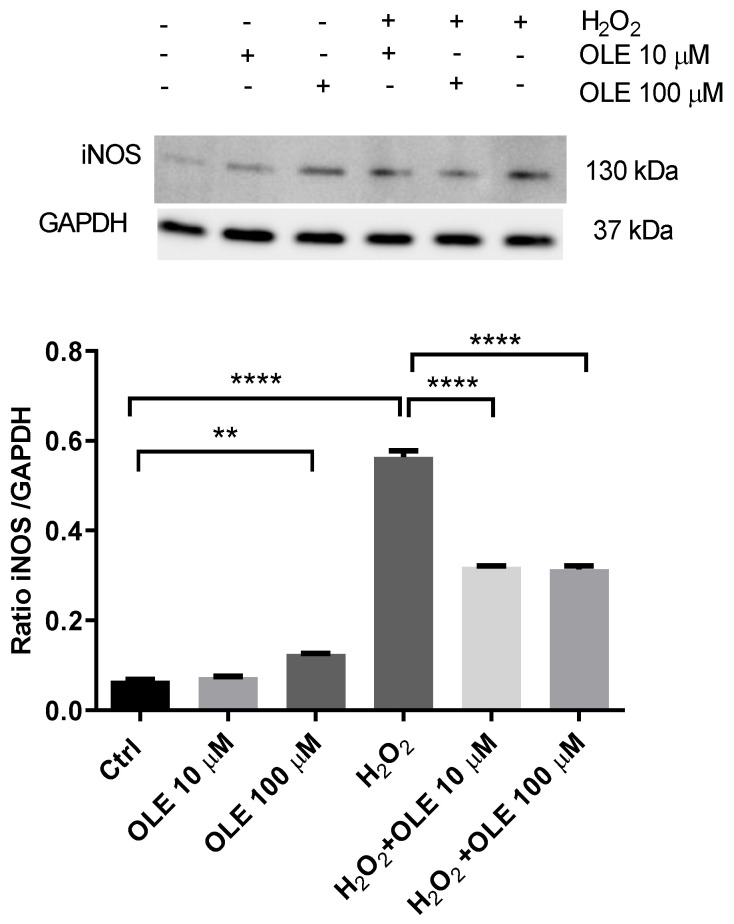
The effect of oleuropein (OLE) on the inducible nitric oxide synthase (iNOS) protein expression in H_2_O_2_-exposed HTR-8/SVneo cells. Upper panel, representative Western blot of iNOS; lower panel, densitometric analysis of iNOS expression in HTR-8/SVneo cells. The data are expressed as mean + SEM. ** *p* < 0.01, **** *p* < 0.0001.

The analysis of OLE’s effects on the inducible nitric oxide synthase (iNOS) protein expression in H_2_O_2_-exposed HTR-8/SVneo cells is represented in Figure 6. The results show that incubation with 10 µM OLE did not alter the levels of iNOS after 24 h incubation, while 100 µM OLE induced a slight but significant increase in exposed trophoblast cells. When cells were treated with H_2_O_2_ only, there was a severe increase in iNOS expression, compared to non-exposed controls. OLE pre-treatment significantly reduced the iNOS protein expression following H_2_O_2_ exposure, displaying an equal effect for both OLE concentrations.

### 3.6. The Effect of Oleuropein on the mRNA Expression of BAX, BCL2 and NFE2L2 in H_2_O_2_-Exposed HTR-8/SVneo Cells

**Figure 7:**
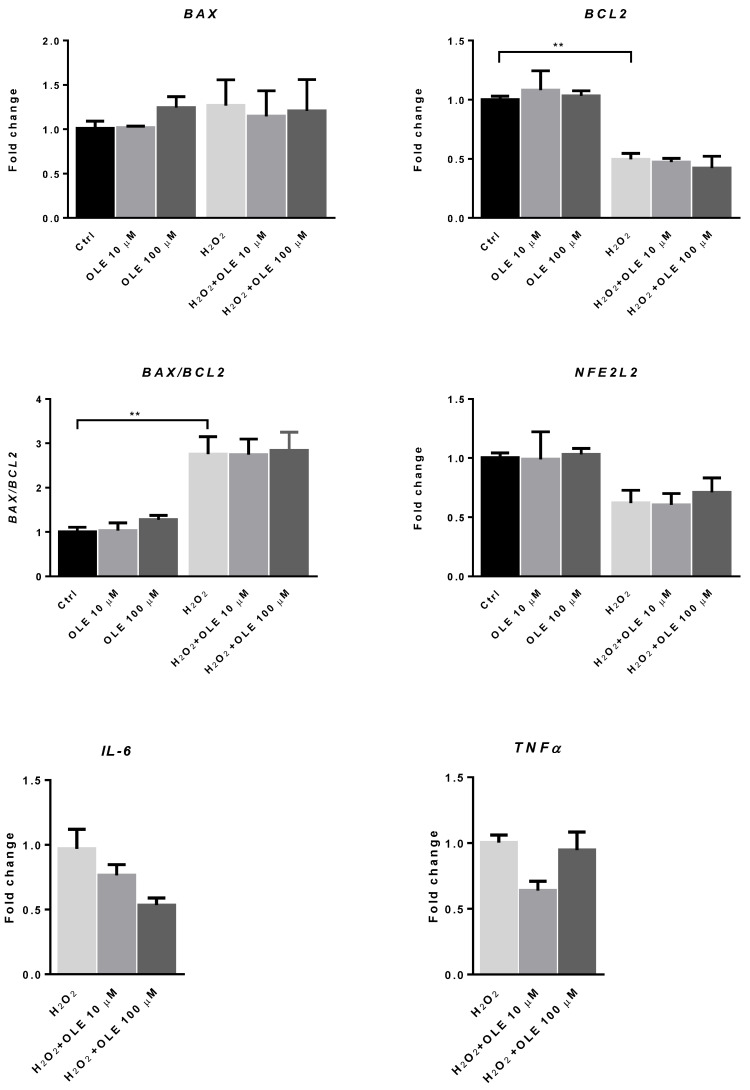
The effect of oleuropein (OLE) on the mRNA expression of apoptotic marker *BAX*, the anti-apoptotic marker *BCL2*, the mRNA ratio of *BAX* to *BCL2* and the mRNA expression of redox-regulated transcription factor *NFE2L2*, interleukin 6 (*IL6*) and tumor necrosis factor α (*TNFα*), in H_2_O_2_-exposed HTR-8/SVneo cells. The data are expressed as mean + SEM. ** *p* < 0.01.

To examine the possible impact of OLE on the mRNA expression of redox-sensitive transcription factor *NFE2L2* and the markers of apoptotic signaling *BAX* and *BCL2*, trophoblast cells were incubated with OLE at 10 and 100 µM for 24 h. Figure 7 shows that treatment with OLE alone did not change the mRNA expression levels of *NFE2L2* or those of *BAX* and *BCL2*. To induce the state of increased OS, we treated HTR-8/SVneo cells with H_2_O_2_ (200 μM) for 2 h, after 24 h exposure to OLE. When the cells were stimulated with H_2_O_2_ alone, the mRNA expression level of the apoptotic marker *BAX* was increased, while the anti-apoptotic *BCL2* mRNA level was significantly decreased compared to non-exposed controls. The *BAX/BCL2* mRNA ratio was significantly increased compared to the non-exposed cells, indicating increased pro-apoptotic signaling in H_2_O_2_-exposed cells. Although the mRNA level of *NFE2L2*, a gene encoding for nuclear factor erythroid 2-related factor 2 (Nrf2), which mediates antioxidant gene upregulation, was reduced in the H_2_O_2_-exposed cells, this decrease did not reach statistical significance. Moreover, OLE pre-treatment did not significantly influence the expression of the examined pro- and anti-apoptotic genes after H_2_O_2_ exposure neither could change the mRNA expression level of *NFE2L2* in trophoblast HTR-8/SVneo cells. In terms of the mRNA expression of pro-inflammatory cytokines *IL-6* and *TNF-α*, without exposure to H_2_O_2_, the levels were low in control cells and unchanged in OLE-treated samples (data not shown). In the cells exposed to H_2_O_2_, the pre-incubation with OLE provided a reduction in the mRNA levels of both cytokines compared to H_2_O_2_, and this reduction was observed for both concentrations of OLE. However, the change was not statistically significant.

## 4. Discussion

Given the importance of ROS during the placentation process and the fact that OS correlates with the severity of some pregnancy-related disorders, such as PE, antioxidant treatment strategies have emerged as an attractive target in clinical trials. However, results derived from clinical trials have been conflicting, and very few antioxidant components showed significant effects in pregnancy disorders [19,35,36,37]. Moreover, most common antioxidant supplements, such as vitamin C and vitamin E, were not only found to be ineffective in reducing the disease risk but have even been associated with increased unexplained stillbirths [38]. These findings suggest that the use of high-dose antioxidants in pregnancy needs to be carefully examined, particularly during the vulnerable early stage of gestation, when interference with oxidative signaling may compromise pregnancy establishment and placental development [39]. Special focus in safety research should be given to the antioxidants that are commonly used in high doses and are readily available throughout various food sources, with the aim to identify the benefits and hazards of their use and evaluate their potential to contribute to the prevention of pregnancy-related disorders [40]. 

OLE is an olive-derived bioactive compound that harbors many health benefits and is characterized by good absorption, being widely distributed in various organs and tissues, reaching them quickly after ingestion [41]. Studies with olive oil supplementation in the pre-conception period and/or during pregnancy demonstrated that a period of supplementation could improve embryo quality parameters in *in vitro* human embryo development, positively affect embryonic growth, modify the epigenetic programming in the placenta and induce an anti-inflammatory environment in the placenta and umbilical cord blood [42,43,44]. Furthermore, OLE supplementation was shown to be effective in alleviating symptoms of GDM and improved gestational outcomes in a mouse model [45]. Moreover, studies in pregnant mice showed that supplementation with extra virgin olive oil could induce the inhibition of oxidative stress pathways [46]. 

It seems that most of the observed health-benefiting effects of olive oil and the derived OLE can be attributed to their antioxidant role and the modulation of OS-related signaling pathways. The results of numerous studies are in support of this assumption. Namely, human trials demonstrated that OLE ingestion could improve the postprandial glycemic profile via hampering NADPH oxidase 2 (Nox2)-derived oxidative stress [47]. It was also shown that OLE exerts its protective role in human liver cells against H_2_O_2_-induced apoptosis via an increase in the expression of antioxidant enzymes SOD1, CAT and GPx 1 [48]. In another study, pre-treatment of human glioblastoma cells (U87) with 10 µM OLE significantly prevented the loss of cells and regenerated the total antioxidant capacity and GSH levels after H_2_O_2_ exposure [49]. This is in line with our results, which showed that in HTR-8/SVneo trophoblast cells exposed to H_2_O_2_, OLE provides the restoration of GSH levels and prevents a decrease in cell viability compromised by H_2_O_2_. OLE also restored the H_2_O_2_-induced activity of the antioxidant enzymes SOD1, CAT and GPx in trophoblast cells, reaching values found in non-treated controls. These findings indicate that OLE significantly improves trophoblast antioxidant defense and the capacity to withstand OS. 

Another significant finding in our study was the decreased levels of MDA and LDH activity following the pre-incubation with OLE and exposure to H_2_O_2_, indicating that OLE is able to reduce the rate of lipid peroxidation in trophoblast cells. This effect was also observed in human embryonic kidney (HEK-293) cells, where OLE treatment led to a marked decrease in lipid peroxidation, as evidenced by reduced MDA production, and prevented H_2_O_2_-induced apoptosis [50]. This is also in line with the observation of García-Villalba et al. [51], who found that decreased MDA levels in plasma (32%) were associated with the consumption of OLE-rich olive leaf extract. In the current study, we also found that both trophoblast iNOS activity and NO end-product nitrites were significantly reduced in cells pre-treated with OLE and challenged with H_2_O_2_. This beneficial effect of OLE is especially important since it is shown that NO plays an important role in regulating trophoblast migration, invasion and apoptosis, which are crucial processes in the establishment of a successful pregnancy [52]. Moreover, increased iNOS activity and NO production are known to directly relate to the severity of PE and are significantly increased in the placentas of patients with this disorder, compared to those with a normal pregnancy [53,54,55]. 

In addition to oxidative signaling, pro-inflammatory cytokines are also known to influence gestational processes, and tumor necrosis factor α (TNF-α) and interleukin 6 (IL-6) are specified as important regulators of placental function [56,57]. Their physiological levels are required in the placenta for proper regulation of the proliferation and differentiation of various types of cells, including trophoblasts [58]. On the other hand, the increased levels of pro-inflammatory cytokines are associated with pregnancy complications, such as recurrent spontaneous abortions, preterm labor, GDM and PE [56,59,60,61,62]. Therefore, a fine-tuned, balanced inflammatory environment in the placenta is crucial for successful pregnancy [63]. In this work, exposure to the higher levels of H_2_O_2_ was used to mimic the state of increased trophoblast OS and induce the expression of the pro-inflammatory cytokines TNF-α and IL-6. The pre-incubation with OLE at 10 µM and 100 µM provided a decrease (although not statistically significant) in TNF-α and IL-6 mRNA expression in H_2_O_2_-exposed trophoblasts, suggesting a possible slight, but direct, anti-inflammatory role for OLE in OS, in addition to its antioxidant properties in placental cells.

The ROS–inflammatory axis in normal pregnancy is balanced by anti-inflammatory mediators, endocrine factors and cell signaling pathways mediating OS. If disturbed, alterations in these processes could lead to various pregnancy pathologies [64]. Nuclear factor erythroid-related factor 2 (Nrf2) is one of the main transcription factors that play a key role in the regulation of the cellular redox balance and the protective antioxidant responses against OS [65]. In the state of a balanced (unstressed) redox environment, Nrf2 is localized mainly in the cytoplasm, but upon the induction of oxidative stress, Nrf2 is translocated in the nucleus, where it upregulates the expression of antioxidant genes [66]. It was previously reported that Nrf2 expression levels, as well as the expression of other Nrf2 target genes, were significantly reduced in PE compared to normal pregnancy placentas [67]. Moreover, under hypoxia/reoxygenation conditions in HTR8/SVneo cells, as well as in the placentas of patients with PE, the activity of OS-related enzymes (CAT, GPx, SOD) were significantly lower, possibly related to the altered levels of the Keap1/Nrf2 signaling pathway [68]. It was also shown that the *in vitro* exposure of the JAR trophoblast cell line to H_2_O_2_ reduced Nrf2 expression, as well as the downstream expression of its target cytoprotective genes. This process was shown to be time-dependent, where significant changes in gene expression could be seen after 6 h [66]. As with the mentioned study, our results showed that incubation with H_2_O_2_ resulted in decreased gene expression of *NFE2L2* and an increased *BAX/BCL2* ratio in mRNA in trophoblast cells. Considering that we used higher concentrations and a shorter (2 h) treatment with H_2_O_2_, the observed changes for *NFE2L2* were most likely not significant due to the short time of exposure. A previous study performed under cyclophosphamide-induced oxidative stress in rats pre-treated with either 100 or 200 mg/kg OLE-rich olive leaf extract showed a marked decrease in *Bax* gene expression and the *Bax/Bcl2* mRNA ratio, and upregulation of the anti-apoptotic *Bcl2* and *Nfe2l2* genes [69]. The same study showed that a dose of 100 mg/kg olive leaf extract remarkably ameliorated the elevated levels of protein carbonyls and nitrites, as well as MDA, in rats with cyclophosphamide-induced oxidative stress. Further, there was a positive effect of olive leaf extract on the antioxidant defense system via increased GSH content and improved activity of the antioxidant enzymes SOD, CAT and GPx [69]. Similar to the mentioned study, our results demonstrated that OLE pre-treatment in HTR-8/SVneo trophoblast cells increased GSH and restored the normal activity of CAT, GPx and SOD following H_2_O_2_ exposure. It also reduced the protein damage and lipid peroxidation induced by OS. However, it could not rescue the decrease in Nrf2 expression and increase in the *BAX/BCL2* mRNA ratio, as well as DNA damage, in trophoblast cells under the current conditions, indicating that the observed positive effect on the antioxidant system could be mediated via pathways other than Nrf2. Previous studies demonstrated cytoprotective effects of OLE against OS in mesenchymal stem cells and human embryonic kidney cells, where it reduced H_2_O_2_-induced apoptosis through modulating MAPK pathways [50,70]. In synovial fibroblasts, OLE treatment provided anti-inflammatory and antioxidant effects via the downregulation of the MAPK and NF-κB signaling pathways, as well as the induction of Nrf2 [71]. Fröhlich et al. provided an important clue about the underlying molecular mechanisms regulating trophoblast survival under elevated OS, by searching for an answer as to why the number of dead trophoblast cells was not affected by the antioxidant treatment, even when the ROS levels were effectively reduced in these cells. Namely, these authors showed that reduced trophoblast proliferation happens even if the antioxidant markers are increased in these cells, in an ROS-independent manner involving the MAPK pathway—specifically, extracellular signal-regulated protein kinase 1 and 2 (ERK1/2) [72]. The activation of the ERK pathway is a component of the cellular damage response that can be initiated by diverse types of exposure, including OS. It also plays an important functional role in cell cycle arrest in response to DNA damage [73]. ERK activation as a response to DNA damage happens quickly, with the first phase of ERK activation starting already at 1 h post-exposure, and it is known to mediate damage-induced apoptosis [74]. A study by Li et al. showed that treatment of cells with 200 µM H_2_O_2_ can simultaneously induce damage by activating the phosphorylation of ERK1/2 and by increasing ROS production and the *BAX/BCL2* ratio, with both pathways leading to the induction of apoptosis [75]. In addition, Go et al. demonstrated that H_2_O_2_’s effects are mediated both directly, by inducing redox signaling through kinase signaling pathways, and indirectly, by oxidizing the cellular environment [76]. This mechanism is distinct from OS and is regulated by a more isolated and localized redox circuitry [76]. The same study also showed that H_2_O_2_ regulates transcription via upstream ERK1/2 and the JNK kinase pathway, separately from the direct regulation of the redox-sensitive transcription factors such as Nrf2. Since our results demonstrated that OLE could not prevent the decrease in *NFE2L2* mRNA expression and the significant increase in DNA damage and the *BAX/BCL2* mRNA ratio, a surrogate marker of pro-apoptotic signaling after H_2_O_2_ exposure [77], it could be assumed that the protective effects of OLE against oxidative damage seen in trophoblast cells in our study may be regulated via the MAPK pathway, which would be interesting to examine in further studies. This assumption is also supported by the fact that the iNOS/NO signaling pathway is triggered by the activation of the ERK signaling pathway in trophoblast cells, as shown by Du and colleagues [78]. Our results showed that OLE treatment effectively reduces iNOS levels following H_2_O_2_ exposure; thus, it is plausible that it also influences the upstream ERK pathway. However, the involvement of other mechanisms behind this protective effect of OLE in trophoblast cells cannot be disregarded. 

## 5. Conclusions

Olive-derived bioactive compound OLE displayed cytoprotective effects against H_2_O_2_ in HTR-8/SVneo trophoblast cells, by increasing antioxidant cell defense, mirrored in the significantly increased expression of antioxidant markers, and the prevention of protein oxidation and lipid peroxidation, as well as by reducing the iNOS levels and the mRNA expression of *IL-6* and *TNFα*. Despite harboring these benefits, OLE did not influence the expression of the redox-sensitive antioxidant transcription factor *NFE2L2*, nor did it prevent the generation of DNA damage or increase pro-apoptotic signaling (according to the increased *BAX/BCL2* mRNA ratio), following H_2_O_2_ exposure. Having in mind the significant attenuation of OS markers and the restoration of antioxidant functioning in HTR-8/SVneo cells pre-treated with OLE, it can be concluded that its protective role could be ascribed to its antioxidant effect, by alleviating the oxidizing cellular environment. However, its impact on the signaling pathways mediating OS in trophoblast cells remains to be examined in further studies. Most importantly, it should be emphasized that OLE per se did not lead to any adverse effects in HTR-8/SVneo trophoblast cells under the described conditions, confirming its safety *in vitro*.

## Data Availability

Data is contained within the article.
